# Fructose co‐ingestion to increase carbohydrate availability in athletes

**DOI:** 10.1113/JP277116

**Published:** 2019-07-02

**Authors:** Cas J. Fuchs, Javier T. Gonzalez, Luc J. C. van Loon

**Affiliations:** ^1^ Department of Human Biology, NUTRIM School of Nutrition and Translational Research in Metabolism Maastricht University Medical Centre+ (MUMC+) Maastricht The Netherlands; ^2^ Department for Health University of Bath Bath UK

**Keywords:** Simple Sugars, Glucose, Sucrose, Oxidation, Glycogen, Liver, Metabolism, Muscle, Resynthesis, Sports Nutrition

## Abstract

Carbohydrate availability is important to maximize endurance performance during prolonged bouts of moderate‐ to high‐intensity exercise as well as for acute post‐exercise recovery. The primary form of carbohydrates that are typically ingested during and after exercise are glucose (polymers). However, intestinal glucose absorption can be limited by the capacity of the intestinal glucose transport system (SGLT1). Intestinal fructose uptake is not regulated by the same transport system, as it largely depends on GLUT5 as opposed to SGLT1 transporters. Combining the intake of glucose plus fructose can further increase total exogenous carbohydrate availability and, as such, allow higher exogenous carbohydrate oxidation rates. Ingesting a mixture of both glucose and fructose can improve endurance exercise performance compared to equivalent amounts of glucose (polymers) only. Fructose co‐ingestion can also accelerate post‐exercise (liver) glycogen repletion rates, which may be relevant when rapid (<24 h) recovery is required. Furthermore, fructose co‐ingestion can lower gastrointestinal distress when relatively large amounts of carbohydrate (>1.2 g/kg/h) are ingested during post‐exercise recovery. In conclusion, combined ingestion of fructose with glucose may be preferred over the ingestion of glucose (polymers) only to help trained athletes maximize endurance performance during prolonged moderate‐ to high‐intensity exercise sessions and accelerate post‐exercise (liver) glycogen repletion.

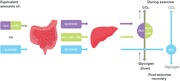

## Graphical abstract

Fructose (Fru) co‐ingestion with glucose (Glu) appears to increase the total capacity to absorb carbohydrates. In addition, fructose can be converted within the intestine and the liver into glucose and lactate (Lac). This can be used as an additional fuel source during exercise and also as a substrate for (liver) glycogen repletion during post‐exercise recovery. Therefore, fructose co‐ingestion may benefit athletes by maximizing carbohydrate availability during exercise and during acute post‐exercise recovery.

## Introduction

Carbohydrates are a major substrate source during prolonged moderate‐ to high‐intensity exercise (Romijn *et al*. 1993; van Loon *et al*. 2001). In the fasted state, the main forms of carbohydrate utilised during exercise are skeletal muscle glycogen and plasma glucose (primarily derived from liver glycogen and gluconeogenesis) (van Loon *et al*. 2001). However, these glycogen stores can be rapidly depleted (by ∼40–60%) within 90 min of moderate to high‐intensity exercise (Casey *et al*. 2000; Stevenson *et al*. 2009; Gonzalez *et al*. 2015). Low endogenous glycogen stores can contribute to fatigue, thereby reducing endurance exercise capacity (Bergstrom *et al*. 1967; Coyle *et al*. 1986; Ortenblad *et al*. 2011; Alghannam *et al*. 2016). Even when glycogen is not critically low (>100 mmol/kg wet wt), higher carbohydrate availability could increase endurance performance by reducing the oxygen cost of exercise as the energy yield per given volume of oxygen is higher from carbohydrate compared to fat‐based fuels (Krogh & Lindhard, 1920). In line with this, it has previously been observed in elite race walkers that both exercise economy and performance were negatively impacted following 3 weeks of a high‐fat diet compared to high carbohydrate availability (Burke *et al*. 2017a). Hence, high carbohydrate availability could provide an advantage during endurance exercise events where oxygen delivery can become a limiting factor. Therefore, nutritional strategies to complement or replace endogenous carbohydrate stores as a fuel source during exercise can be of importance for athletes trying to maximize endurance exercise performance. It is now well established that carbohydrate ingestion during exercise improves endurance performance and can delay fatigue in events requiring sustained moderate‐ to high‐intensity exercise for prolonged durations (i.e. more than ∼45 min; Currell & Jeukendrup, 2008; Vandenbogaerde & Hopkins, 2011).

Due to the apparent relationship between glycogen depletion and endurance exercise capacity (Casey *et al*. 2000; Alghannam *et al*. 2016), an important determinant of recovery time is the rate of glycogen repletion. This is particularly relevant when optimal performance needs to be regained well within 24 h, for example during intensive training periods, tournament‐style competitions or in between stages in multiday races such as the Tour de France. In the hours following exercise, carbohydrate ingestion is a requirement for substantial repletion of liver and skeletal muscle glycogen stores (Ivy *et al*. 1988; Casey *et al*. 2000; van Hall *et al*. 2000).

Dietary carbohydrates come in many forms, with glucose (polymers) being the most ubiquitous carbohydrate in most people's diets (Gonzalez *et al*. 2017). Glucose is also the primary cellular fuel source in most human tissues. As a result, glucose (polymers) have been recommended already for decades as the predominant source of carbohydrates to ingest around endurance exercise sessions for athletes (Hawley *et al*. 1992). Fructose, on the other hand, has long since been considered a suboptimal source of carbohydrate to ingest around exercise, as it seems less effective at increasing exogenous carbohydrate oxidation (compared to glucose) and may cause gastrointestinal distress (Convertino *et al*. 1996). More recently, there has been an increasing appreciation of ingesting a combination of glucose and fructose both during and after exercise. Therefore, this review provides a brief overview of the potential benefits of (co‐)ingesting fructose with glucose (polymers) during exercise and acute post‐exercise recovery.

## Carbohydrate ingestion during exercise

During exercise, exogenous carbohydrate oxidation rates differ depending on the type of carbohydrate that is consumed (Cermak & van Loon, 2013). It has been well established that the maximal exogenous carbohydrate oxidation rate increases in a curvilinear fashion with carbohydrate ingestion rate, reaching peak exogenous oxidation rates of ∼1.1 g/min when ingesting glucose (polymers) only during exercise (Jeukendrup & Jentjens, 2000; Gonzalez *et al*. 2017). Several factors may determine the rate at which exogenous carbohydrates are taken up and oxidized by the working muscles during exercise. These include the rate of gastric emptying, the rate of digestion and absorption, passage via the liver into the systemic blood supply, and the rate of glucose uptake and subsequent oxidation by the working muscle (Jeukendrup, 2004). The primary limitation of exogenous carbohydrate oxidation rates is unlikely to be caused by gastric emptying rates, as gastric emptying rates of glucose have been shown to exceed carbohydrate oxidation rates during prolonged exercise (Rehrer *et al*. 1992). In addition, the primary limitation of exogenous carbohydrate oxidation rates is also unlikely to be caused by glucose uptake and oxidation by the working muscle, as when glucose is directly infused (thereby bypassing the intestines and liver), peak exogenous carbohydrate oxidation rates of 1.8 g/min can be achieved (Hawley *et al*. 1994). This implies that intestinal absorption and/or hepatic metabolism may be the primary factors limiting exogenous glucose oxidation rate during exercise (Jeukendrup & Jentjens, 2000; Rosset *et al*. 2017a).

When (only) fructose is ingested, exogenous carbohydrate oxidation rates during exercise have been shown to be equivalent (Decombaz *et al*. 1985; Massicotte *et al*. 1990; Burelle *et al*. 2006) or lower compared to glucose ingestion (Massicotte *et al*. 1986, 1989, 1990; Guezennec *et al*. 1989; Jandrain *et al*. 1993; Adopo *et al*. 1994; Burelle *et al*. 1997). Furthermore, ingestion of large amounts of fructose (alone) has been reported to cause gastrointestinal distress, and the capacity for intestinal absorption of fructose ingested alone has also been shown to be limited (Truswell *et al*. 1988; Fujisawa *et al*. 1993). Consequently, fructose has generally been considered of little interest for the athlete trying to optimize carbohydrate availability during exercise (Jeukendrup & Jentjens, 2000; Cermak & van Loon, 2013). However, when fructose is co‐ingested with glucose during exercise (at 50% maximum power output (*W*_max_)), exogenous carbohydrate oxidation rates can increase up to 1.75 g/min (Jentjens & Jeukendrup, 2005), which is substantially higher than the rates reported following ingestion of glucose (polymers) or fructose only. These exogenous carbohydrate oxidation rates do not appear to differ when fructose is co‐ingested in the form of fructose or sucrose (Trommelen *et al*. 2017), which is in line with observations that rates of digestion and intestinal absorption of glucose and fructose do not differ whether they are ingested as sucrose or co‐ingested as free fructose and free glucose (Gray & Ingelfinger, 1966).

**Figure 1:**
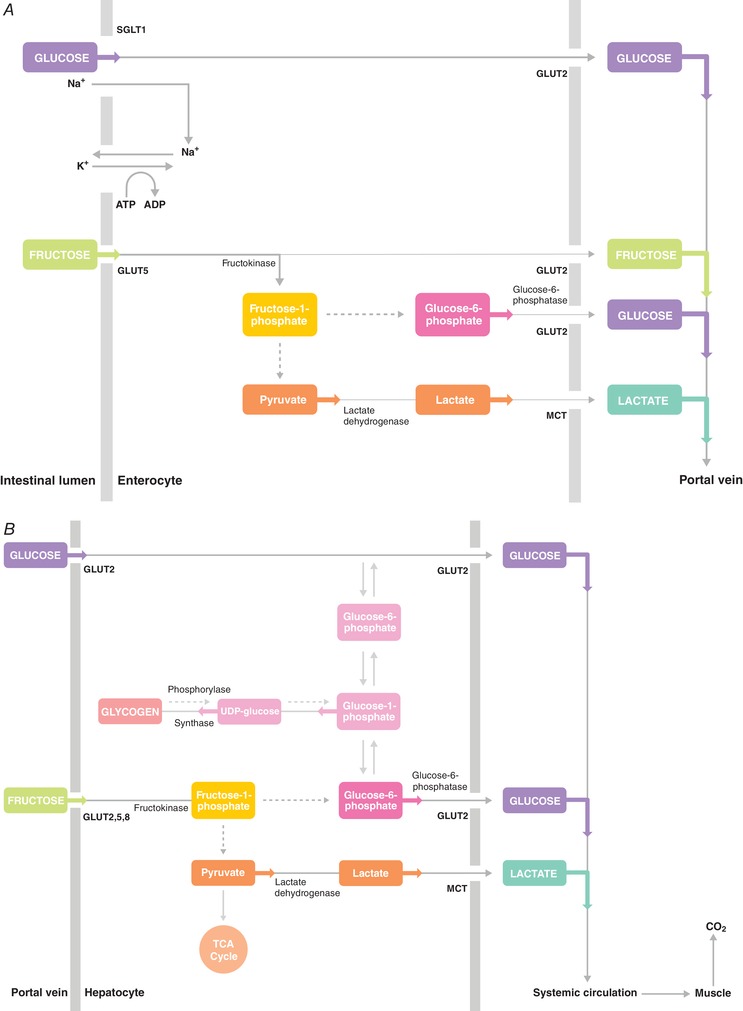
Main pathways involved in intestinal (*A*) and hepatic (*B*) glucose and fructose absorption and fructose conversion into glucose and lactate *A*, intestinal glucose and fructose absorption and fructose conversion into glucose and lactate within the enterocytes. Glucose is primarily absorbed via the sodium dependent glucose transporter 1 (SGLT1) and is largely transmitted passively into the portal vein. Fructose is primarily absorbed via glucose transporter 5 (GLUT5) and can be transmitted into the portal vein via glucose transporter 2 (GLUT2) or can be metabolized within the enterocyte. Via first conversion into fructose‐1‐phosphate (via fructokinase) glucose‐6‐phosphate and pyruvate can be formed. Glucose‐6‐phosphate can be converted into glucose (via glucose‐6‐phosphatase) and leave the enterocyte via GLUT2. Pyruvate can be converted into lactate (via lactate dehydrogenase) and leave the enterocyte via monocarboxylate transporter (MCT). *B*, main pathways involved in hepatic glucose and fructose absorption and fructose conversion into glucose and lactate during exercise. Glucose is primarily taken up via GLUT2 and is largely transmitted passively into the systemic circulation. Fructose can be taken up via GLUT2, GLUT5 and/or GLUT8 and is largely metabolized into glucose and lactate during exercise. Via first conversion into fructose‐1‐phosphate (via fructokinase) glucose‐6‐phosphate and pyruvate can be formed. Glucose‐6‐phosphate can be converted into glucose (via glucose‐6‐phosphatase) and leave the hepatocyte via GLUT2. Glucose‐6‐phosphate can also be used as a substrate for restoring liver glycogen during exercise (via conversion first into glucose‐1‐phosphate and subsequently into UDP‐glucose). However, fructose co‐ingestion is not more effective in preventing liver glycogen depletion during exercise compared to glucose ingestion only, suggesting that liver glycogen storage during exercise may not be a primary pathway. Pyruvate can be used as substrate to provide direct energy to the liver (TCA cycle) or can be converted into lactate (via lactate dehydrogenase) and leave the hepatocyte via the monocarboxylate transporter (MCT). The additional glucose and lactate (derived from fructose) can be used as substrate for oxidation in the muscle during exercise. GLUT, glucose transporter; TCA Cycle, tricarboxylic acid cycle; UDP‐glucose, uridine diphosphate glucose.

Fructose metabolism differs markedly from glucose metabolism. At rest, fructose is primarily absorbed across the apical membrane of the intestinal enterocytes by transport protein GLUT5, whereas glucose is primarily absorbed across the apical membrane of the intestinal enterocytes by transport protein SGLT1 (please see Ferraris *et al*. (2018) for a comprehensive review on this topic). After intestinal absorption, fructose appears to be actively metabolized in the splanchnic area (i.e. intestine, liver and kidneys), whereas glucose appears to be largely transmitted passively from the splanchnic area into the systemic circulation (Gonzalez *et al*. 2017; Tappy & Rosset, 2017; Jang *et al*. 2018; Tappy, 2018; Fig. 1). Therefore, following fructose ingestion, plasma fructose concentrations remain relatively low (<0.5 mmol/l) (Lecoultre *et al*. 2010; Rosset *et al*. 2017b) as fructose is rapidly converted in the intestine and liver to glucose and lactate (Fig. 1), which then enter the systemic circulation and are delivered to peripheral tissues (Lecoultre *et al*. 2010) and/or contribute to liver glycogen synthesis (Gonzalez *et al*. 2016). It should be noted that recent work has reported that ∼15% of ingested fructose may escape first‐pass extraction by the splanchnic organs (Francey *et al*. 2019) and, as such, may be directly metabolized in other tissues (Tappy, 2018). However, direct oxidation of fructose in the muscle is unlikely to play a significant quantitative role as fuel during exercise since fructose transport capacity over the muscle membrane is 8 times lower than glucose and this capacity is not further increased by exercise (Kristiansen *et al*. 1997). In line with this, the lower affinity of hexokinase for fructose compared to glucose further supports the notion that plasma fructose is not an important fuel source for exercising muscle (Rikmenspoel & Caputo, 1966). When fructose is co‐ingested with glucose in large amounts (0.8 g/min and 1.2 g/min, respectively) during exercise, the systemic appearance of fructose‐derived glucose and lactate is ∼0.5 g/min (of which the contribution of glucose and lactate is equally split) (Lecoultre *et al*. 2010). The oxidation of fructose‐derived glucose and lactate by skeletal muscle can thus fully account for the higher exogenous carbohydrate oxidation rates observed following ingestion of glucose and fructose mixtures compared to glucose only (Gonzalez *et al*. 2017).

In addition to providing fuel to the working muscle, ingesting carbohydrates during exercise has also been suggested to prevent the depletion of endogenous (muscle and liver) carbohydrate stores. Due to the higher capacity for carbohydrate absorption and the predominant hepatic metabolism of fructose (Fig. 1), it could be speculated that the combined ingestion of glucose and fructose further prevents the lowering of endogenous carbohydrate stores during exercise, particularly in the liver. We recently performed a study investigating the effects of ingesting either 1.7 g/min of glucose or 1.7 g/min of sucrose during 3 h of exercise (at 50% *W*_peak_) on liver and muscle glycogen concentrations (Gonzalez *et al*. 2015). We observed that neither type of carbohydrate was able to prevent the lowering of muscle glycogen concentrations during exercise. However, ingestion of either type of carbohydrate fully prevented liver glycogen depletion. This suggests (at least during submaximal exercise at 50% *W*_peak_) that carbohydrate ingestion can prevent liver glycogen depletion during exercise, but that fructose co‐ingestion is not more effective than glucose (polymers) ingestion alone in preventing exercise‐induced glycogen depletion. Whether the same holds true for exercise sessions performed at higher exercise intensities remains to be elucidated.

Increases in plasma glucose and insulin concentrations inhibit net hepatic glycogenolysis (Petersen *et al*. 1998). We have observed that plasma glucose and insulin concentrations do not differ during moderate intensity exercise (at 50% *W*_peak_) when fructose and glucose are co‐ingested compared to ingesting glucose only (Gonzalez *et al*. 2015; Trommelen *et al*. 2017). These findings seem to be in line with the absence of differences in liver glycogen concentrations when either glucose or sucrose was ingested (Gonzalez *et al*. 2015). When large amounts of carbohydrate are ingested during exercise, the co‐ingestion of fructose lowers gastrointestinal distress compared to ingesting equivalent amounts of glucose (polymers) alone (Jentjens *et al*. 2004; de Oliveira *et al*. 2014; Gonzalez *et al*. 2015; Trommelen *et al*. 2017). Therefore, the main benefits of fructose co‐ingestion (*vs*. glucose (polymers) only) during exercise are due to increased exogenous (and total) carbohydrate oxidation rates and/or less gastrointestinal discomfort, rather than preventing muscle or liver glycogen depletion. It has been observed that athletes co‐ingesting glucose (polymers) and fructose, compared to ingesting glucose (polymers) only, can further improve endurance exercise performance by ∼8–9% (this was found when glucose‐fructose mixtures (≥90 g/h) were compared to equivalent amounts of glucose as well as amounts of glucose (60 g/h) that are proposed to saturate intestinal absorption) (Currell & Jeukendrup, 2008; Triplett *et al*. 2010; Stellingwerff & Cox, 2014; King *et al*. 2018). It is important to note, however, that ingestion of large amounts (>1.2 g/min) of a mixture of glucose (polymers) and fructose is likely to be only of practical relevance to highly trained athletes that are able to sustain high‐intensity exercise for a prolonged duration (i.e. > 2.5 h) (Jeukendrup, 2014).

## Carbohydrate ingestion after exercise

The suggestion that glucose‐fructose co‐ingestion will increase rates of carbohydrate absorption also raises the possibility of further accelerating the rate of endogenous (muscle and/or liver) carbohydrate stores during recovery from exercise. It has been hypothesized that greater carbohydrate availability through ingestion of large amounts of glucose and fructose (sucrose) mixtures could, therefore, augment post‐exercise glycogen repletion rates.

### Muscle glycogen

It has previously been demonstrated that net muscle glycogen (re)synthesis rates during 4 h of post‐exercise recovery in the fasted state are in the range of ∼2–12 mmol/kg dw/h (Maehlum & Hermansen, 1978; Ivy *et al*. 1988; van Hall *et al*. 2000), with no net muscle glycogen (re)synthesis observed beyond 4 h of recovery (Maehlum & Hermansen, 1978). In the first few hours (∼4 h) after exercise, skeletal muscle glycogen (re)synthesis rates are enhanced due – at least in part – to an increase in insulin sensitivity (Richter *et al*. 1982). With sufficient carbohydrate intake immediately post‐exercise, net muscle glycogen (re)synthesis rates have been observed to increase up to 20–45 mmol/kg dw/h (Beelen *et al*. 2010), and with frequent carbohydrate ingestion (alongside increased insulin availability) this can result in a full recovery of muscle glycogen levels within 24 h (Burke *et al*. 2017b; Gonzalez & Betts, 2019). For more in‐depth information on muscle glycogen repletion, the interested reader is referred to other reviews (e.g. Jentjens & Jeukendrup (2003), Beelen *et al*. (2010) and Burke *et al*. (2017b). It has been well established that for optimal post‐exercise net muscle glycogen (re)synthesis rates, athletes should ingest carbohydrates at a rate of ∼1.2 g/kg/h immediately after cessation of exercise and in frequent intervals (i.e. 15–30 min) within the first ∼4 h of the recovery period (Burke *et al*. 2017b). With regard to the type of carbohydrate ingested, it has previously been observed that glucose ingestion increases post‐exercise muscle glycogen repletion rates more than fructose ingestion only (Blom *et al*. 1987; Van Den Bergh *et al*. 1996). However, it has been speculated that based on the metabolism of glucose and fructose during exercise (Fig. 1), greater carbohydrate availability following ingestion of large amounts of glucose plus fructose could further increase post‐exercise muscle glycogen repletion rates compared to ingestion of glucose only. Several studies have directly compared ingestion of mixtures of glucose (polymers) with fructose and glucose (polymers) alone on post‐exercise muscle glycogen repletion rates (Blom *et al*. 1987; Bowtell *et al*. 2000; Casey *et al*. 2000; Wallis *et al*. 2008; Fuchs *et al*. 2016; Trommelen *et al*. 2016). In these studies a wide range of carbohydrate ingestion rates have been employed ranging from 0.25 to 1.5 g/kg body mass/h over 2–6 h of recovery. Based on these studies it can be concluded that even with large, recommended carbohydrate ingestion rates (≥1.2 g/kg/h) provided at frequent intervals, there are no differences between the effects of ingestion of glucose (polymers) and fructose (sucrose) mixtures *vs*. glucose (polymers) alone on post‐exercise muscle glycogen repletion rates (Wallis *et al*. 2008; Fuchs *et al*. 2016; Trommelen *et al*. 2016). However, the ingestion of large amounts (≥1.2 g/kg/h) of glucose and fructose mixtures have been shown to result in lower gastrointestinal issues, probably due to improved intestinal carbohydrate absorption (Fuchs *et al*. 2016; Trommelen *et al*. 2016). This is a relevant finding as gastrointestinal distress could directly reduce the capacity to perform optimally in a subsequent bout of exercise.

### Liver glycogen

In contrast to muscle, the liver plays a major role in fructose metabolism and is able to synthesize glucose from fructose in meaningful quantities. Over a 6‐h period, up to ∼50% of ingested fructose can be found in the circulation as glucose, the conversion of which seems to occur primarily in the liver. In addition, there is some conversion of fructose into glucose that is subsequently stored directly as liver glycogen, which seems to account for at least > 15% of fructose disposal at rest. (Tappy & Le, 2010; Sun & Empie, 2012). Consequently, fructose co‐ingestion may further accelerate liver glycogen repletion compared to the ingestion of glucose (polymers) only.

**Figure 2:**
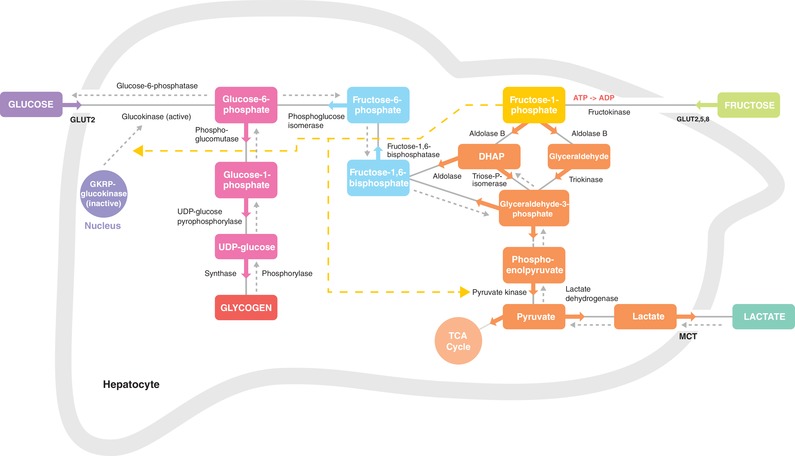
Proposed hepatic fructose metabolism into glucose, glycogen and lactate after exercise Upon entering hepatocytes, fructose is phosphorylated by fructokinase to fructose‐1‐phosphate. Fructose‐1‐phosphate is cleaved to DHAP and glyceraldehyde by aldolase B. DHAP and glyceraldehyde can be phosphorylated (by triose‐P‐isomerase and triokinase, respectively) into glyceraldehyde‐3‐phosphate. Both DHAP and glyceraldehyde‐3‐phosphate can enter the gluconeogenic and/or glycolytic metabolite pool, and can have several metabolic fates including conversion into glucose, glycogen and lactate. Fructose‐1‐phosphate (shown in yellow) also regulates metabolic enzymes (yellow lines) involved in glycogen storage and lactate production. Responsible enzymes denoted in black; responsible transporters denoted in black and bold. DHAP, dihydroxyacetone phosphate; GKRP, glucokinase regulatory protein; GLUT, glucose transporter; MCT, monocarboxylate transporter; TCA cycle, tricarboxylic acid cycle; Triose‐P‐isomerase, triose‐phosphate‐isomerase; UDP, uridine diphosphate.

Upon intestinal absorption, fructose can be metabolized within the small intestine (albeit most likely only in small amounts in humans, as saturation of intestinal fructose metabolism has been suggested to occur at ∼5 g of fructose intake) (Tappy & Le, 2010; Tappy & Rosset, 2017; Jang *et al*. 2018; Gonzalez & Betts, 2018), or transported to the liver via the portal vein (Fig. 1). Fructose uptake in the liver is thought to be mainly operated by the glucose transporter GLUT2, but GLUT5 and GLUT8 may also contribute (Hannou *et al*. 2018). Within the liver, fructose is largely extracted at first pass and rapidly phosphorylated into fructose‐1‐phosphate by the enzyme fructokinase (also known as ketohexokinase), which is highly specific for fructose. Fructose‐1‐phosphate is subsequently metabolized into glyceraldehyde and dihydroxyacetone phosphate (DHAP) via aldolase B. Subsequently, glyceraldehyde‐3‐phosphate can be formed (via the enzymes triose‐phosphate‐isomerase and triokinase), which can be further converted (first via fructose‐1,6‐bisphosphate and fructose‐6‐phosphate) into glucose‐6‐phosphate (see Fig. 2 for schematic overview). Within the liver, glucose‐6‐phosphate can be converted into glucose (by glucose‐6‐phosphatase) and subsequently released into the systemic circulation (e.g. to maintain euglycaemia) or stored (via conversion first into glucose‐1‐phosphate and subsequently into UDP‐glucose) as liver glycogen.

In addition to providing an indirect substrate for liver glycogen synthesis, fructose‐1‐phosphate can also act as a signalling molecule to further increase liver glycogen synthesis. Fructose‐1‐phosphate exerts a strong positive regulatory control on glucokinase by promoting its release from the inhibitory glucokinase regulatory protein (GKRP) (Van Schaftingen *et al*. 1994; McGuinness & Cherrington, 2003; Agius, 2008). In the fasted state, GKRP sequesters glucokinase in the nucleus in an inactive state (Agius, 2008). Via activation of glucokinase, small amounts of fructose can promote hepatic glucose uptake and phosphorylation (into glucose‐6‐phosphate), leading to rapid glycogen synthesis (Shiota *et al*. 1998; Petersen *et al*. 2001; McGuinness & Cherrington, 2003) (Fig. 2). Within the liver, elevated glucose‐6‐phosphate concentrations provoke the activation of glycogen synthase (Villar‐Palasi & Guinovart, 1997) and Petersen *et al*. (2001) demonstrated that low‐dose fructose (∼9 g) infusion during a 4 h hyperinsulinaemic‐euglycaemic clamp further increased liver glycogen synthase flux (by ∼2.5‐fold) and subsequent net glycogen synthesis rates (by ∼3‐fold) in healthy young humans. It should be noted that insulin potentiates hepatic glycogen synthesis by activating glycogen synthase and inactivating glycogen phosphorylase (Hers, 1976; Aiston *et al*. 2003) and intravenous fructose administration during a hyperinsulinaemic‐euglycaemic clamp is not necessarily reflective of oral fructose (co‐)ingestion in the post‐exercise state. Previous studies have observed that post‐exercise fructose‐glucose co‐ingestion (when ingested regularly in small boluses) can induce a robust increase in insulin concentrations that remain elevated during post‐exercise recovery (Decombaz *et al*. 2011; Fuchs *et al*. 2016). In fact, similar insulin concentrations can be observed when fructose is co‐ingested (Decombaz *et al*. 2011) compared to intravenous fructose administration during a hyperinsulinaemic‐euglyacemic clamp (Petersen *et al*. 2001). However, despite increased insulin concentrations (compared to basal values) and a doubling of post‐exercise liver glycogen repletion rates (compared to ingesting glucose only), the increase in plasma insulin concentrations with fructose co‐ingestion is typically still lower than the increase seen with ingestion of glucose only during post‐exercise recovery (Fuchs *et al*. 2016). In addition, even in the absence of increased plasma insulin, the administration of small amounts of intravenous fructose (∼2.2 g) with glucose markedly increases hepatic glycogen content, compared to glucose only, in dogs (Shiota *et al*. 2005). Thus, it appears that even small ‘catalytic’ doses of fructose added to glucose can augment liver glucose uptake and glycogen synthesis, which cannot be attributed to changes in plasma insulin *per se*. The latter may be explained (at least in part) by the fact that the production of fructose‐1‐phosphate from fructose and activation of glucokinase by fructose‐1‐phosphate are not dependent on increased plasma insulin levels (Agius & Peak, 1993; Van Schaftingen *et al*. 1994). Fructose‐1‐phosphate has also been suggested to augment hepatic glycogen storage by inhibiting glycogen phosphorylase (Van Den Berghe *et al*. 1973; Thurston *et al*. 1974). However, Petersen *et al*. (2001 did not show an inhibitory effect of fructose on glycogen phosphorylase flux in human subjects, suggesting that this pathway may not contribute to augmented liver glycogen storage when fructose is administered in humans.

Finally, fructose‐1‐phosphate can activate pyruvate kinase (Eggleston & Woods, 1970), thereby contributing to increased circulating levels of lactate following fructose (co‐)ingestion (Fig. 2). Indeed, we (Fuchs *et al*. 2016; Trommelen *et al*. 2016) and others (Wallis *et al*. 2008; Rosset *et al*. 2017b) have previously observed greater post‐exercise lactate concentrations following fructose‐glucose co‐ingestion, compared to glucose (polymers) only. Lactate has been shown to serve as an additional substrate source, but can also be transported to the muscle to directly stimulate muscle glycogen repletion (Hermansen & Vaage, 1977; Astrand *et al*. 1986; Bangsbo *et al*. 1991, 1997; Medbo *et al*. 2006). Lactate may also indirectly stimulate muscle glycogen repletion, via conversion within the liver (by gluconeogenesis) into glucose and subsequent transport to the muscle. These effects of lactate could potentially explain (at least in part) equivalent muscle glycogen repletion with fructose co‐ingestion (*vs*. glucose alone), as fructose appears to retain some of the (exogenous) glucose within the hepatocytes. Finally, lactate can also indirectly contribute to hepatic glycogen synthesis (Brooks, 2018).

Via these mechanisms, in addition to higher rates of carbohydrate absorption and availability, fructose co‐ingestion could theoretically further accelerate liver glycogen synthesis rates over glucose ingestion only. A few studies have directly compared the effects of glucose and fructose co‐ingestion *vs*. glucose (polymer) ingestion only on post‐exercise liver glycogen repletion rates (Moriarty *et al*. 1994; Casey *et al*. 2000; Decombaz *et al*. 2011; Fuchs *et al*. 2016). Only two of these studies provided carbohydrates at relatively high ingestion rates (>0.9 g/kg body mass/h) that have been recommended for optimal post‐exercise glycogen repletion in athletes (Decombaz *et al*. 2011; Fuchs *et al*. 2016). Based on these studies, it can be concluded that when glucose is ingested alone, the rate of post‐exercise liver glycogen repletion is ∼3.5 g/h. When fructose is co‐ingested with glucose (either as free glucose and fructose or as sucrose), the rate of liver glycogen repletion may increase 2‐fold (∼7.4 g/h) (Decombaz *et al*. 2011; Fuchs *et al*. 2016). Therefore, it can be concluded that the combined ingestion of glucose plus fructose accelerates liver glycogen repletion.

It has been suggested that fructose co‐ingestion can increase endurance exercise capacity by an additional 3–5 min during a subsequent bout of cycling exercise (at 75% *W*_max_) due to its capacity to accelerate liver glycogen repletion (Fuchs *et al*. 2016). In support of this, when fructose was co‐ingested during the first 4 h of post‐exercise recovery, endurance exercise capacity during a subsequent exercise bout (i.e. treadmill running to exhaustion at 70% V˙O2 max ) was shown to be increased by ∼32.4% compared to ingesting glucose (polymers) only (Maunder *et al*. 2018). This clearly shows the potential benefit of combining glucose with fructose in the first hours of post‐exercise recovery when optimal performance during a subsequent endurance exercise session is key.

It should be noted that high dietary fructose intake has been proposed to induce adverse health effects and has been associated with the development of metabolic disease (Hannou *et al*. 2018; Tappy, 2018). Therefore, more research in the area of fructose‐rich diets and their potential adverse effects are warranted. However, exercise appears to be able to correct early markers of metabolic disease induced by high fructose intake, independent of energy balance (Egli *et al*. 2013; Wilburn *et al*. 2015). In addition, elite athletes typically display exquisite metabolic health, as demonstrated by a 3‐fold higher insulin sensitivity than controls (Manetta *et al*. 2000) despite consuming large amounts (>450 g per day) of simple sugars during events such as the Tour de France (Saris *et al*. 1989). Therefore, given the ergogenic properties of fructose both during and after exercise, athletes may benefit from fructose co‐ingestion.

## Conclusions and recommendations

The rate of appearance of ingested glucose in the circulation appears to be limited by the capacity of intestinal transporters. Since intestinal fructose absorption utilises a different transport mechanism, combining the ingestion of fructose and glucose takes advantage of both transport mechanisms, thereby increasing the total capacity for carbohydrate absorption. This can be beneficial during exercise to further increase exogenous carbohydrate oxidation rates and decrease gastrointestinal discomfort when large amounts of carbohydrates are ingested, thereby improving endurance exercise performance during prolonged moderate‐to‐high intensity exercise. Consequently, when trying to maximize performance, well‐trained athletes are advised to combine the ingestion of glucose and fructose at ingestion rates of 90 g/h during prolonged (>2.5 h) moderate‐to‐high intensity exercise. After exercise, rapid recovery of both muscle and liver glycogen stores are important determinants of the capacity to perform a subsequent bout of moderate‐to‐high intensity exercise. Muscle glycogen repletion rates cannot be further increased with fructose‐glucose co‐ingestion. However, probably due to its higher absorption rate and/or the predominant hepatic metabolism of fructose, fructose co‐ingestion has been observed to enhance post‐exercise liver glycogen repletion rates without compromising muscle glycogen re‐synthesis. In addition, when large amounts of carbohydrates are ingested after exercise, the combined ingestion of glucose plus fructose can result in less gastrointestinal distress. Therefore, when rapid recovery from prolonged exercise is a key objective, and maximal performance is required well within 24 hours, it is advised to consume more than 1 g carbohydrate/kg body mass/h, starting as soon as possible after exercise and at frequent intervals thereafter (i.e. every 15–30 min). In this context, fructose co‐ingestion may be of benefit to lower gastrointestinal discomfort and accelerate liver glycogen synthesis rates.

## Additional information

### Competing interests

No competing interests declared.

### Author contributions

All authors have approved the final version of the manuscript and agree to be accountable for all aspects of the work. All persons designated as authors qualify for authorship, and all those who qualify for authorship are listed.

### Funding

No sources of funding were used to prepare this manuscript. L.J.C.vL. has received research support from Pepsico and Kenniscentrum Suiker en Voeding. J.T.G. has received research support from Arla Foods Ingredients, Lucozade Ribena Suntory and Kenniscentrum Suiker en Voeding.
